# Reaction Time and Visual Memory in Connection to Hazardous Drinking Polygenic Scores in Schizophrenia, Schizoaffective Disorder and Bipolar Disorder

**DOI:** 10.3390/brainsci11111422

**Published:** 2021-10-27

**Authors:** Atiqul Haq Mazumder, Jennifer Barnett, Erkki Tapio Isometsä, Nina Lindberg, Minna Torniainen-Holm, Markku Lähteenvuo, Kaisla Lahdensuo, Martta Kerkelä, Ari Ahola-Olli, Jarmo Hietala, Olli Kampman, Tuula Kieseppä, Tuomas Jukuri, Katja Häkkinen, Erik Cederlöf, Willehard Haaki, Risto Kajanne, Asko Wegelius, Teemu Männynsalo, Jussi Niemi-Pynttäri, Kimmo Suokas, Jouko Lönnqvist, Jari Tiihonen, Tiina Paunio, Seppo Juhani Vainio, Aarno Palotie, Solja Niemelä, Jaana Suvisaari, Juha Veijola

**Affiliations:** 1 Department of Psychiatry, Research Unit of Clinical Neuroscience, University of Oulu, 90014 Oulu, Finland; Martta.Kerkela@oulu.fi (M.K.); Tuomas.Jukuri@oulu.fi (T.J.); Juha.Veijola@oulu.fi (J.V.); 2 Cambridge Cognition, University of Cambridge, Cambridge CB25 9TU, UK; jhb32@cam.ac.uk; 3 Department of Psychiatry, University Hospital and University of Helsinki, 00029 Helsinki, Finland; erkki.isometsa@helsinki.fi (E.T.I.); nina.lindberg@helsinki.fi (N.L.); Tuula.kieseppa@hus.fi (T.K.); asko.wegelius@fimnet.fi (A.W.); tiina.paunio@helsinki.fi (T.P.); 4 Mental Health Unit, Finnish Institute for Health and Welfare (THL), 00271 Helsinki, Finland; minna.torniainen-holm@thl.fi (M.T.-H.); erik.cederlof@thl.fi (E.C.); jouko.lonnqvist@thl.fi (J.L.); jaana.suvisaari@thl.fi (J.S.); 5 Department of Forensic Psychiatry, Niuvanniemi Hospital, University of Eastern Finland, 70240 Kuopio, Finland; Markku.Lahteenvuo@niuva.fi (M.L.); Katja.Hakkinen@niuva.fi (K.H.); jari.tiihonen@ki.se (J.T.); 6 Institute for Molecular Medicine Finland (FIMM), University of Helsinki, 00014 Helsinki, Finland; kaisla.lahdensuo@icloud.com (K.L.); ari.ahola-olli@helsinki.fi (A.A.-O.); hawker@utu.fi (W.H.); risto.kajanne@helsinki.fi (R.K.); teemu.mannynsalo@hel.fi (T.M.); jussi.niemi-pynttari@hel.fi (J.N.-P.); kimmo.suokas@tuni.fi (K.S.); aarno.palotie@helsinki.fi (A.P.); 7 Mehiläinen, Pohjoinen Hesperiankatu 17 C, 00260 Helsinki, Finland; 8 Department of Psychiatry, University of Turku, 20014 Turku, Finland; jarmo.Hietala@tyks.fi (J.H.); solnie@utu.fi (S.N.); 9 Department of Psychiatry, Turku University Hospital, 20521 Turku, Finland; 10 Faculty of Medicine and Health Technology, Tampere University, 33014 Tampere, Finland; olli.kampman@tuni.fi; 11 Department of Psychiatry, Pirkanmaa Hospital District, 33521 Tampere, Finland; 12 Social Services and Health Care Sector, City of Helsinki, 00099 Helsinki, Finland; 13 Department of Psychiatry, University of Helsinki, 00014 Helsinki, Finland; 14 Department of Clinical Neuroscience, Karolinska Institute, 17177 Stockholm, Sweden; 15 Center for Psychiatry Research, Stockholm City Council, 11364 Stockholm, Sweden; 16 Infotech Oulu, University of Oulu, 90014 Oulu, Finland; seppo.vainio@oulu.fi; 17 Northern Finland Biobank Borealis, Oulu University Hospital, 90220 Oulu, Finland; 18 Faculty of Biochemistry and Molecular Medicine, University of Oulu, 90014 Oulu, Finland; 19 Kvantum Institute, University of Oulu, 90014 Oulu, Finland; 20 Stanley Center for Psychiatric Research, The Broad Institute of MIT (Massachusetts Institute of Technology) and Harvard, Cambridge, MA 02142, USA; 21 Analytical and Translational Genetics Unit, Massachusetts General Hospital, Boston, MA 02114, USA; 22 Department of Psychiatry, Oulu University Hospital, 90220 Oulu, Finland

**Keywords:** cognition, visual memory, reaction time, hazardous drinking, PGS, schizophrenia, schizoaffective disorder, bipolar disorder

## Abstract

The purpose of this study was to explore the association of cognition with hazardous drinking Polygenic Scores (PGS) in 2649 schizophrenia, 558 schizoaffective disorder, and 1125 bipolar disorder patients in Finland. Hazardous drinking PGS was computed using the LDPred program. Participants performed two computerized tasks from the Cambridge Automated Neuropsychological Test Battery (CANTAB) on a tablet computer: the 5-choice serial reaction time task, or Reaction Time (RT) test, and the Paired Associative Learning (PAL) test. The association between hazardous drinking PGS and cognition was measured using four cognition variables. Log-linear regression was used in Reaction Time (RT) assessment, and logistic regression was used in PAL assessment. All analyses were conducted separately for males and females. After adjustment of age, age of onset, education, household pattern, and depressive symptoms, hazardous drinking PGS was not associated with reaction time or visual memory in male or female patients with schizophrenia, schizoaffective, and bipolar disorder.

## 1. Introduction

Polygenic Score (PGS) is the combined measure of a larger number of risk genes for any disease. The PGS for alcohol has been defined in many ways according to what kind of alcohol use or diagnosis has been studied and also according to which genes have been studied considerably [1,2].

In normal population studies, PGS for alcohol dependence has been found to be negatively associated with cognitive function [3,4]. No evidence was found for a causal association of cognitive impairment for rs1229984 in Alcohol Dehydrogenase 1B (ADH1B) [5,6] (Almeida et al. 2014, Kumari M et al. 2014) or rs671 in Alcohol Dehydrogenase 2 (ALDH2) [7] in the normal population [8]. Light to moderate alcohol use has been found to be associated with a decreased risk of cognitive impairment [9] in the normal population. However, this J-shaped relationship between alcohol use and cognition could be attributed to potential abstainer errors [10,11,12,13] and reverse causality bias [14].

Alcohol use PGS has been found to be associated with alcohol use, alcohol-related morbidity, and all-cause mortality [15]. PGS for alcohol use disorder has been found to be positively associated with alcohol use [4]. PGS for the problem sub-scale of Alcohol Use Disorders Identification Test (AUDIT-P) has been found to be associated with alcohol use disorder [16]. PGS for drinks per week has also been found to be positively associated with drinks per week [17]. Hazardous alcohol use PGS has been found to be associated with hazardous alcohol use in our study samples.

To our knowledge, no previous studies have focused on the association of alcohol PGS and cognition in schizophrenia, schizoaffective, and bipolar disorder patients.

The main aim of the present study was to explore the association of reaction time and visual memory with hazardous drinking PGS in people with schizophrenia, schizoaffective, and bipolar disorder. Processing speed and visual learning are two important features of cognitive domains invariably affected in several neuropsychiatric conditions [18]; hence, we selected the Five-Choice Serial Reaction Time Task (5-CRTT) and the Paired Associative Learning (PAL) task from the Cambridge Neuropsychological Test Automated Battery (CANTAB) for the assessment of Reaction Time (RT) and visual memory, respectively.

The specific research aims were to study the following:

1. The association of hazardous drinking PGS with reaction time and visual memory in schizophrenia patients;
2. The association of hazardous drinking PGS with reaction time and visual memory in schizoaffective disorder patients;
3. The association of hazardous drinking PGS with reaction time and visual memory in bipolar disorder patients.

## 2. Materials and Methods

### 2.1. Application of STROBE (Strengthening the Reporting of Observational Studies in Epidemiology) Checklist for Cross-Sectional Studies

STROBE checklist for cross-sectional studies has been applied in this study and is attached as a supplementary document for reference (https://www.strobe-statement.org/download/strobe-checklist-cross-sectional-studies-pdf, accessed on 23 October 2021).

### 2.2. Study Design

The study design was a cross-sectional study of a representative sample of people with psychotic disorders encompassing the whole of Finland. The SUPER study aimed to collect genotype and phenotype information from at least 10,000 Finnish patients suffering from psychotic mental illness.

A DNA sample acquired via phlebotomy (or saliva, if the blood sample was not feasible) from every study subject went through Whole-Genome Sequencing (WGS). The genomic data were then be analyzed in association with the phenotype data collected (questionnaire, interview, blood sample analysis (plasma/serum, cells, biomarkers, and RNA), and registry data).

### 2.3. Timetable

The pilot study was commenced in the fall of 2015 and ended in 2016. The main study started in 2016 and continued for three years (2016–2018).

### 2.4. Participant Number Estimation

The minimum number of participants for effective analysis was estimated to be around 10,000. Permission was sought to continue sample collection for the whole three-year sample collection period as long as the budget holds. The aim was achieved, and 10,417 samples were collected.

### 2.5. Sample Collection Strategy

The sample collection was divided between five university hospital districts that would organize the local sample collections. This way, an optimal collection strategy was possible to adjust for each region based on their demographic and geographical requirements. The patients were directly identified from the patient flow during the routine clinic, and the patient recruitment and sample collection were initiated without delay once approved by the ethical committee. Permission to use hospital district registers and private healthcare contractor registers to identify and contact suitable patients using diagnostic codes were sought from the respective authorities.

### 2.6. Exclusion Criteria

(1) Being <18 years (underage);

(2) Being ≥70 years;

(3) Being unable to provide informed consent as evaluated by the trained research personnel or attending physician;

(4) Not living independently;

(5) Missing information on education;

(6) Missing information on depressive symptoms;

(7) Missing information on hazardous drinking PGS.

### 2.7. Missing Data Handling

For this study, we excluded participants who had missing data on hazardous drinking PGS, education, and depressive symptoms.

### 2.8. Data Handling

The nationwide collection of samples required a centralized information system to store the data and to effectively hold a record of patients who had been contacted, who had already participated, and who had declined in order to avoid duplications and contacting the same individuals’ multiple times. For this reason, a study register was formed at FIMM (the Finnish Institute for Molecular Medicine). A secure online submission system for phenotype data was created for the study. All data were encoded for analysis. Once the recruitment and registry data collection were finished, the participants’ personal data (name, address, and social security number) were deleted from the phenotype database, and the participants were identified only with their study ID. The key code linking participants’ study ID with their personal data was stored in a locked, hall monitored data system at the FIMM. The DNA samples, blood samples, and phenotype data collected were ultimately stored at the THL (Terveyden ja Hyvinvoinnin Laitos-The Finnish Institute for Health and Welfare) biobank.

### 2.9. Data Storage

After sample collection, the data were processed and analyzed, which might likely take several years to complete. Permission to store samples and data by the research group was initially for 20 years, with the possibility to apply for 10-year extensions if needed.

### 2.10. Sensitivity Analyses

We have performed sensitivity analyses also including those participants aged 70 years and above.

### 2.11. Participants

The participants of this study were part of the 10,417-study population of the Suomalainen psykoosisairauksien perinnöllisyysmekanismien tutkimus study (“Finnish Study of the Hereditary Mechanisms behind Psychotic Illnesses”—SUPER), which was part of the international Stanley Global Neuropsychiatric Genomics Initiative. SUPER collected data from five university hospital districts in Finland during the period 2016–2019 from people with the lifetime diagnosis of psychotic illnesses, as classified by ICD-10 diagnostic codes F20–F29 (F20 Schizophrenia, F21 Schizotypal disorder, F22 Persistent delusional disorders, F23 Acute and transient psychotic disorders, F24 Induced delusional disorder, F25 Schizoaffective disorders, F28 Other nonorganic psychotic disorders, F29 Unspecified nonorganic psychosis), F30.2 (Mania with psychotic symptoms), F31.2 (Bipolar affective disorder, current episode manic with psychotic symptoms), F31.5 (Bipolar affective disorder, current episode severe depression with psychotic symptoms), F32.3 (Severe depressive episode with psychotic symptoms), and F33.3 (Recurrent depressive disorder, current episode severe with psychotic symptoms), to identify gene loci and gene variations predisposing patients to psychotic illnesses and comorbid diseases. These codes were used to identify subjects from the Care Register for Health Care (CRHC) and in clinical settings. In the CRHC, upon fulfilling the diagnostic criteria for psychiatric disorders, clinical diagnoses were made by clinicians, mostly in specialized care by psychiatrists. In Finland, the International Classification of Disease (ICD) system is used in psychiatric diagnoses. ICD-8 was used from 1968 to 1986 and ICD-9 from 1987 to 1995, while ICD-10 has been used since 1996 in Finland. During the use of ICD-9 in Finland (1987–1995), DSM-3 R criteria for bipolar disorder and other psychiatric disorders had been used.

In clinical settings, such as healthcare centers, nursing homes, and psychiatric treatment facilities, staff were asked to select patients with these diagnoses to be voluntarily recruited into the SUPER study. Subjects were also recruited via advertisements in local newspapers. Underage patients and patients unable to provide informed consent as evaluated by the trained research personnel or attending physician were excluded from the study.

**Figure 1:**
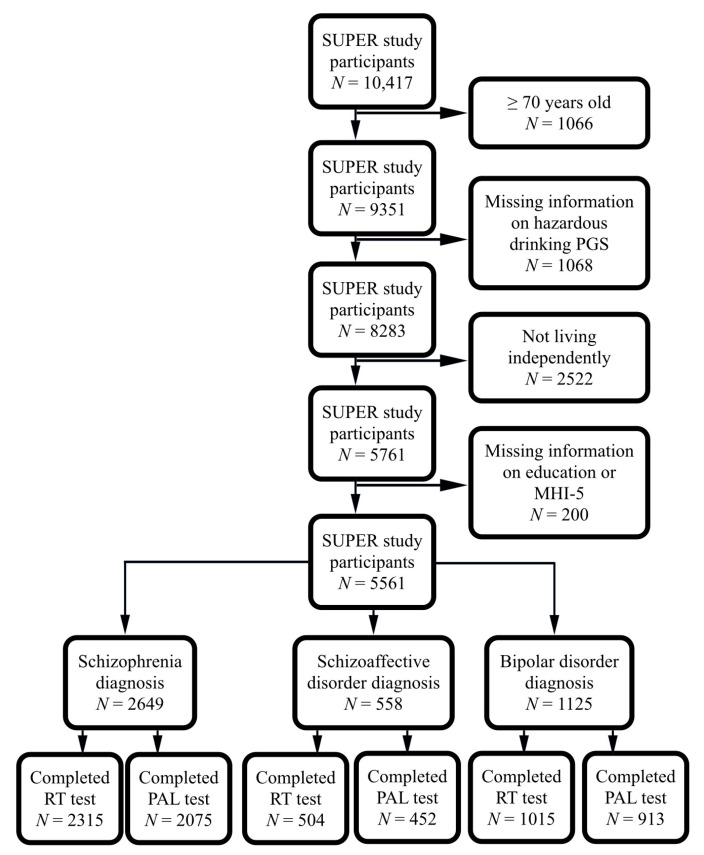
Flowchart showing the selected study populations. SUPER, Suomalainen psykoosisairauksien perinnöllisyysmekanismien tutkimus; MHI-5, Mental Health Inventory-5; RT, Reaction Time; PAL, Paired Associative Learning.

Out of the original 10,417 study participants, we included 2649 with a lifetime diagnosis of schizophrenia (1639 only schizophrenia, 101 schizophrenia + bipolar disorder, 664 schizophrenia + schizoaffective disorder, 245 schizophrenia + schizoaffective disorder + bipolar disorder), 558 with a lifetime diagnosis of schizoaffective disorder (299 only schizoaffective disorder, 259 schizoaffective + bipolar disorder) and 1730 with a lifetime diagnosis of only bipolar disorder (without schizophrenia or schizoaffective disorder), and excluded those aged 70 years and above, not living independently and with missing information on alcohol PGS, education or MHI-5. Considering the hierarchy of severity of illness, duel diagnosed participants were included in schizophrenia and schizoaffective disorder diagnosis groups. Among the schizophrenia diagnosis group, 2315 (1252 males, 1063 females) completed the Reaction Time (RT) test, and 2075 (1124 males, 951 females) completed the Paired Association Learning (PAL) test. Among the schizoaffective disorder diagnosis group, 504 (174 males, 330 females) completed the Reaction Time (RT) test, and 452 (159 males, 292 females) completed the Paired Association Learning (PAL) test. Among the bipolar disorder diagnosis group, 1015 (369 males, 646 females) completed the RT test, and 913 (322 males, 591 females) completed the PAL test. Among the combined study population group, 3850 (1799 males, 2051 females) completed the RT test, and 3440 (1605 males, 1835 females) completed the PAL test (Figure 1 and Supplementary Figure S1).

### 2.12. Schizophrenia Diagnoses

The diagnosis of schizophrenia was obtained from the CRHC. In this study, schizophrenia diagnoses included codes 295, according to ICD-8 and ICD-9, and F20, according to ICD-10.

### 2.13. Schizoaffective Disorder Diagnoses

In this study, schizoaffective disorder diagnoses included codes 295.7, according to ICD-8 and ICD-9, and F25, according to ICD-10.

### 2.14. Bipolar Disorder Diagnoses

In this study, bipolar disorder diagnoses included both mania and bipolar disorder corresponding to the codes 296.1–296.8, 298.10 according to ICD-8; 296.2–296.4, 296.7A according to ICD-9 and F30, F31 according to ICD-10.

While selecting the study population, those who were not able to sign the written informed consent themselves were excluded, and those living independently were included. Thus, by far, all hospitalized patients were excluded. Thus, the most severe bipolar patients with severe depressive or manic episodes were presumed to be excluded. However, cognitive impairment due to bipolar disorder in the general population is not limited to the acute hospitalized episodes where patients might be unable to provide informed consent.

### 2.15. Hazardous Drinking Polygenic Scores

We used the LDpred_inf.py software for calculating polygenic risk scores using GWAS summary statistics as training data, which assumes that there is a proportion “p” of SNP variants that were causal. The priority was estimated from the SNP heritability calculated from the summary stats and the causal fraction specified by the user of the program before the calculation. The distribution of polygenic scores is shown in Supplementary Figure S2, and correlations of polygenic scores are shown in Supplementary Excel Spreadsheet S1. The correlations are relatively low (color-coded as the highest correlation red and lowest blue).

The SUPER samples were genotyped with Illumina and Affymetrix arrays (Thermo Fisher Scientific, Santa Clara, CA, USA) for 1.3 million Single Nucleotide Polymorphisms (SNPs) augmented by imputed common HapMap (2003) SNPs, which have been the basis for Genome-Wide Association Studies (GWASs) [19]. Genotype calls were made with GenCall and zCall algorithms for Illumina and AxiomGT1 algorithm for Affymetrix chip genotyping data. Genotyping data produced with previous chip platforms were lifted over to build version 38 (GRCh38/hg38) following the protocol described here: dx.doi.org/10.17504/protocols.io.nqtddwn. Samples with sex discrepancies, high genotype missingness (>5%), excess heterozygosity (±4SD), and non-Finnish ancestry were removed. Variants with high missingness (>2%), deviation from HWE (*p* < 1 × 10^−6^), and low Minor Allele Count (MAC < 3) were removed. Pre-phasing of genotyped data was performed with Eagle 2.3.5 (https://data.broadinstitute.org/alkesgroup/Eagle/, accessed on 23 October 2021) with the default parameters, except the number of conditioning haplotypes was set to 20,000. Imputation was carried out by using the population-specific SISu v3 imputation reference panel with Beagle 4.1 (version 08Jun17.d8b, https://faculty.washington.edu/browning/beagle/b4_1.html, accessed on 23 October 2021) as described in the following protocol: dx.doi.org/10.17504/protocols.io.nmndc5e. SISu v3 imputation reference panel was developed using the high-coverage (25–30x) whole-genome sequencing data generated at the Broad Institute of MIT and Harvard and at the McDonnell Genome Institute at Washington University, and jointly processed at the Broad Institute. Variant callset was produced with GATK HaplotypeCaller algorithm by following GATK best practices for variant calling. Genotype-, sample-, and variant-wise QC was applied in an iterative manner by using the Hail framework v0.1 (https://github.com/hail-is/hail, accessed on 23 October 2021). The resulting high-quality WGS data were phased with Eagle 2.3.5 as described above. Post-imputation quality control involved excluding variants with INFO score <0.7.20. Hazardous drinking PGSs were derived by weighting the individual SNPs by their effect sizes from published GWASs and by polygenic and clinical risk scores and their impact on the age of onset and prediction of hazardous drinking.

Individuals with non-European ancestry or obscure sex were excluded. Quality Control (QC) before phasing and imputation excluded variants with missingness > 5%, call rate < 95%, Minor Allele Count (MAC) < 3 (if Zcalled) or MAC < 10 (if called using Illumina GenCal), INFO < 0.8, minor allele frequency < 0.001%, Hardy–Weinberg equilibrium *p*-value < 1 × 10^−10^ and heterozygosity exceeding ±4 standard deviations. The QC was performed simultaneously on all data. Prior to imputation, the haplotypes were estimated using SHAPEIT2. (1) Imputation was performed with IMPUTE2 (2) using high-coverage, population-specific reference panels of 2690 whole-genome and 5093 whole-exome sequences. For imputed SNPs, the association analysis was a logistic regression of disease state on the expected fractional allele dosage, with seven dummy variables representing the eight strata entered as covariates. The Wald statistic for the dosage coefficient was the primary test statistic. We followed the protocol described in Kiiskinen T et al. 2020 and Vilhjálmsson BJ et al. 2015 [15,20].

### 2.16. Cognitive Measures

As we have already mentioned, processing speed and visual learning might be among the most affected cognitive features in major psychiatric disorders such as schizophrenia, schizoaffective, and bipolar disorder [18]. For this reason, from the CANTAB, we selected the 5-CRTT to assess processing speed in terms of reaction time and the PAL test to assess visual learning and memory.

These tasks were chosen to produce relevant information on cognition in psychotic disorders in the very restricted assessment schedule. The instructions for both tests were translated into Finnish. The CANTAB tests were performed before venipuncture in order to avoid malfunction of the arm due to pain or bandaging. The study nurses were given standardized instructions on how to guide the study subjects in performing the CANTAB test beforehand.

In the RT test, we used two continuous measurements: the median of the five-choice reaction time and the Standard Deviation (SD) of the five-choice reaction time. The median of the five-choice reaction time is the median duration between the onset of the stimulus and the release of the button. The standard deviation of the five-choice reaction time is the standard deviation of the time taken to touch the stimulus after the button has been released. Both variables were calculated for correct, assessed trials where the stimulus could appear in any of the five locations.

In the PAL test, we assessed visual memory using the primary outcome variables of “total errors adjusted” and first trial memory score. First Trial Memory Score (FTMS) is how many patterns the participant correctly places on the first attempt at each problem, while Total Errors (Adjusted) (TEA) reflects how quickly the participant learns when the participant has multiple attempts at each problem. For PAL TEA, we assessed a dichotomized variable because the distribution of the PAL TEA does not follow any known distribution with multiple peaks, using data from Northern Finland Birth Cohort 1966 (NFBC 1966) as a reference data. The NFBC 1966 consists of all born with an expected date in the year 1966. The data used in this study consist of a 46-year follow-up when cohort members took the PAL test during a clinical examination (N = 5608). Scores for total errors adjusted of NFBC66, the 50th percentile (10 error score or less) was used as a cutoff for suitable performance in PAL test in the recent study, meaning the SUPER study population made better error score than a 50% of NFBC 1966 study population. The PAL FTMS variable was used as a continuous variable.

### 2.17. Confounding Factors

Age, age of onset, education [21], housing status [22], and depressive symptoms [23] have effects on cognition. Hence, we considered them to be the confounding variables in this study.

#### 2.17.1. Age

Cognition is negatively associated with increased age in healthy populations [24] and debatably in alcohol users [25]. The age of the participants was calculated using the participation date and year of birth of the participant. Age was used as a continuous variable.

#### 2.17.2. Age of Onset

Illness duration of schizophrenia, schizoaffective, and bipolar disorder, and late-onset bipolar disorder is associated with more severe cognitive impairments [26,27,28,29]; hence, we have used age of onset as a confounding factor. We have checked the multicollinearity effects among “Age” and “Age of onset”, and as the Variance Inflation Factor (VIF) was <5 in all models, so the multicollinearity was not a problem. The “Age” and “Age of onset” were correlated with each other, but the correlation was acceptable.

#### 2.17.3. Education

Education is strongly associated with cognitive performance [30]. The question and possible answers addressing the education of the participants were as follows: “What is your basic education?” (1 = less than primary school, 2 = matriculation examination, 3 = middle school, 4 = partial general upper secondary school or general upper secondary education certificate, 5 = partial middle school or primary school less than nine years, 6 = primary school, 7 = four-year elementary school). During the analysis, we combined classes 1, 3, 4, 5, 6, and 7 as “No matriculation examination” versus class 2 (“Matriculation examination”).

It would be more informative if we could categorize education into three groups. However, it might be difficult for a general reader to understand the diverse categories in the Finnish education system reflecting changes over the past seventy years, plus additional categories reflecting the small proportion of immigrants who might have lower general education than that provided in the Finnish education system. We used the general education variable because the youngest participants could still be students.

#### 2.17.4. Household Pattern

Household patterns, especially living without a spouse, might affect cognition [24,31], and thus we considered household patterns as a confounder. The questions and possible answers addressing household patterns of the participants were: “What is your living style?” (1 = Alone, 2 = With children without spouse, 3 = With parents or siblings, 4 = With spouse, 5 = With spouse and children). During the analysis, we combined classes 1, 2, and 3 as “Without spouse” and classes 4 and 5 as “With spouse”.

#### 2.17.5. Depressive Symptoms

Depressive symptoms might be associated with poorer cognitive performance [23]; hence, we considered depression as a confounder. We used the five-item Mental Health Inventory-5 (MHI-5) to detect depressive symptoms. In the analysis, MHI-5 was dichotomized. We used a ≤72 cutoff score for depression, which was also used in a recent population-based study in Finland [32].

### 2.18. Statistical Methods

We chose the hazardous drinking PGS based on their correlation with hazardous drinking in our study population. The correlation was calculated with a point-biserial correlation.

We evaluated the association between cognition and hazardous drinking PGS by using four different cognition variables: median and standard deviation of RT, PAL FTMS, and PAL TEA. Association between RT test and hazardous drinking PGS was analyzed with log-linear regression, and eβ with 95% Confidence Intervals (CI) were reported. Association between PAL FTMS-test and hazardous drinking PGS was analyzed with linear regression, and β with 95% CI were reported. Association between PAL TEA and hazardous drinking PGS was analyzed with logistic regression, and Odds Ratios (OR) with 95% CI were reported.

All continuous variables used in these models were normalized using a z-score. We assessed crude models and models adjusted with age, education, household pattern, and depressive symptoms. All the analyses were conducted separately for males and females. Previous studies showed sex differences in selective cognitive test performances [33,34]. Effect size measures were also calculated; R^2^ in linear regression and Cohen’s d measurement in logistic regression were reported.

## 3. Results

### 3.1. Background Factors and Hazardous Drinking PGS in Male and Female Schizophrenia Patients

About 54% of participants were males and 46% females. The mean age was 45 years for males and 47 for females. The mean age of onset was 26 years for males and 27 for females. About one-third of the males and two-fifths of the females had the highest basic educational of 12 years (matriculation). About 90% of males and 75% of females were living without a spouse. About two-thirds of participants had depressive symptoms. Most of the participants were on psychotropic medication. About 95% of them were taking antipsychotics, 25% benzodiazepines, about one-third antidepressants, and about one-fifth mood stabilizers. Hazardous drinking PGS was 7.95 × 10^−7^ for males and 7.63 × 10^−7^ for females (Table 1).

### 3.2. Background Factors and Hazardous Drinking PGS in Male and Female Schizoaffective Disorder Patients

About one-third of participants were males and two-third females. The mean age was 42 years for males and 43 for females. The mean age of onset was 30 years. More than two-fifths of participants had the highest basic educational of 12 years (matriculation). About four-fifths of males and two-thirds of females were living without a spouse. More than two-thirds of participants had depressive symptoms. Most of the participants were on psychotropic medication. A total of 93%–94% of them were taking antipsychotics, about one-third benzodiazepines, one-third males and two-fifth females were taking antidepressants, two-fifths males and one-third females were taking mood stabilizers. Hazardous drinking PGS was 7.71 × 10^−7^ for males and 7.77 × 10^−7^ (9.27 × 10^−7^) for females (Table 2).

### 3.3. Background Factors and Hazardous Drinking PGS in Male and Female Bipolar Disorder Patients

Two-fifth of the participants were males and three-fifth females. The mean age was 45 years for males and 44 for females. The mean age of onset was 37 years for males and 36 for females. About one-third of the males and half of the females had the highest basic educational of 12 years (matriculation). About three-fifth of participants were living without a spouse. About 70% of participants had depressive symptoms. About two-fifths of males and one-fourth of females were screened positive for hazardous drinking. Most of the participants were on psychotropic medication. Hazardous drinking PGS was 7.81 × 10^−7^ for males and 8.17 × 10^−7^ for females (Table 3).

Background factors and hazardous drinking PGS in male and female schizophrenia, schizoaffective, and bipolar disorder patients have been shown in Supplementary Table S1.

### 3.4. Association of Hazardous Drinking PGS with RT Test and PAL Test in Male and Female Schizophrenia Patients

There was no statistically significant association, whether positive or negative, between hazardous drinking PGS and reaction time or visual memory in male and female schizophrenia and schizoaffective disorder patients, after adjustment with age, age of onset, education, household pattern, and depressive symptoms (Table 4).

### 3.5. Association of Hazardous Drinking PGS with RT Test and PAL Test in Male and Female Schizoaffective Disorder Patients

There was no statistically significant association, whether positive or negative, between hazardous drinking PGS and reaction time or visual memory in male and female schizoaffective disorder patients, after adjustment with age, age of onset, education, household pattern, and depressive symptoms (Table 5).

### 3.6. Association of Hazardous Drinking PGS with RT Test and PAL Test in Male and Female Bipolar Disorder Patients

There was no statistically significant association, whether positive or negative, between hazardous drinking PGS and reaction time or visual memory in male and female bipolar disorder patients, after adjustment with age, age of onset, education, household pattern, and depressive symptoms (Table 6).

Association of hazardous drinking PGS with RT test and PAL test in male and female schizophrenia, schizoaffective, and bipolar disorder patients have been shown in Supplementary Table S2.

We have performed sensitivity analyses adding 373 participants with the age of 70 years and above, including 241 schizophrenia patients, 36 schizoaffective disorder patients, and 96 bipolar disorder patients, and obtained no statistically significant differences.

## 4. Discussion

### 4.1. Main Findings

Our findings did not support our assumption that hazardous drinking PGS might be associated with impaired cognitive function in terms of reaction time and visual memory in a large sample of schizophrenia, schizoaffective, and bipolar disorder outpatients below 70 years old, living individually.

### 4.2. Comparison with Other Studies

As per our knowledge, there are no other studies investigating the association between cognitive testing in terms of reaction time and visual memory and hazardous drinking PGS in schizophrenia, schizoaffective or, disorder patients; hence, it was difficult to compare our findings with other studies. Most of the studies investigating the cognitive impact of alcohol in persons with bipolar disorder with comorbid AUD revealed a correlation between alcohol use and cognitive impairment.

One study on the genetic overlap between cognition and alcohol dependence in a normal population revealed that PGS for alcohol dependence was negatively associated with phonemic verbal fluency and vocabulary. The same study also revealed that socially deprived people carried more alcohol dependence risk alleles, which might contribute to the increased prevalence of problem drinking [4].

Genetic studies revealed no evidence for a causal association of cognitive impairment for two candidate genetic biomarkers for alcohol dependence, namely rs1229984 in ADH1B [5,6] and rs671 in ALDH2 [7] in the normal population [8]. However, rs1229984 in ADH1B was found to be an association with alcohol consumption in the mothers [35].

PGS for Bipolar Disorder (BD) in persons with BD has been found to be associated with increased risk for cognitive deficits in the children of those persons [36]. PGS for BD has also been associated with altered brain activities while performing emotional and cognitive tasks [37,38,39,40].

Cross-sectional studies in a normal population revealed that the association of moderate to heavy drinking with cognition was negative [41,42,43,44], and the association of mild to moderate drinking with cognition was positive [44,45,46,47,48] or had no association [49,50]. These relations might be non-linear, whereas the current analyses focused on linear relations between PGS and cognitive performance.

As our samples are large, if anything overpowered, the null findings would appear to be meaningful, even if the statistical approach could only lead to a conclusion of failing to reject the null. Our findings would initiate further research to discover unknown variables of gene-environment interaction.

### 4.3. Strengths

The STROBE Checklist for cross-sectional studies has been used to make sure that this research was conducted according to a standardized guideline.

We used a large data set comprising schizophrenia, schizoaffective, and bipolar disorder outpatients to investigate the association between hazardous drinking PGS and cognition. We used age, age of onset, and education as potential confounding variables. We checked multicollinearity among “Age” and “Age of onset” before using age of onset as a confounder. Cognition was assessed with two standard cognition measurement tools. Genetic analysis was conducted precisely following standard protocol.

We included all schizophrenia, schizoaffective, and bipolar disorder outpatients living independently and excluded those whose living circumstances (living in supported housing, hospitals, or unknown residence) might affect their alcohol use. We also confounded household patterns (living with spouse versus without spouse).

Our inclusion criterion of independent living excluded hospitalized patients, so patients with severe manic or depressive episodes were not included. Another inclusion criterion was the ability to provide written informed consent, which also restricted the inclusion of bipolar disorder patients with severe manic or depressive symptoms.

We exclude people aged 70 years and above to minimize reverse causality bias. However, we performed sensitivity analyses keeping those aged 70 years and above but found almost no differences. Similarly, we also analyzed our data excluding age of onset as confounders but obtained almost similar results. The number of participants taking antipsychotics, mood stabilizers, and/or benzodiazepines have been reported. All analyses were conducted separately in males and females. We also analyzed data using alcohol use disorder PGS along with hazardous drinking PGS, but the results were the same “no association”. We also performed Cohen’s d measure of effect size for our study findings.

### 4.4. Limitations

Our study was cross-sectional, not longitudinal. We did not adopt a more comprehensive approach to measure working memory performance. We used only a reaction time test and a memory test from the CANTAB, while most of the literature we reviewed showed that alcohol use in psychotic patients was associated with executive function deficits. So, patients with hazardous drinking might be more impulsive and less accurate. Furthermore, memory impairment is expected to occur only in patients with severe alcohol-related cognitive impairment (formerly known as alcohol dementia), a sub-group of patients with AUD.

We did not use information about heritability [51,52,53], the onset of alcohol use, any recent changes in drinking habits, or any previous history of abstinence. We also did not differentiate previous alcohol users from never-alcohol users and did not exclude individuals who reduced drinking due to illness/doctor’s advice, which might attribute the results through reverse causality bias [54,55]. We did not correct self-report bias [56,57] and Misreports and Longitudinal Changes (MLC), which could affect the study results [58,59]. We did not confound household income, which, as indicative of socioeconomic status, could increase alcohol-related mortality and morbidity despite lower reportedly alcohol consumption (alcohol harm paradox) [60]. We confounded education, which is another strong indicator of socioeconomic status, in a dichotomous fashion, not in a stratified one.

We did not confound antipsychotic medication because almost all of the persons with bipolar disorder were on antipsychotic medication. We did not confound benzodiazepines use as it could impair cognitive performance because of its acute sedative effect. We could not show chlorpromazine equivalent and/or diazepam equivalents, despite those might affect neurocognition. We also did not confound smoking or other substance use during a lifetime, and we did not confound other F1 diagnoses. We did not incorporate Mendelian randomization to minimize possible reverse causality bias. We did not use continuous variables for the PAL test.

We categorized education as completed general secondary education with matriculation examination versus lower. It would be more informative if we could categorize education into three groups. However, it might be difficult for a general reader to understand the diverse categories in the Finnish education system reflecting changes over the past 70 years, plus additional categories reflecting the small proportion of immigrants who might have lower general education than that provided in the Finnish education system. We used the general education variable because the youngest participants could still be students.

Current drinking status might be associated with cognitive changes [61]. It would be valuable to investigate, to the degree possible, whether the cognitive effect was mediated by actual alcohol (ab)use or alcohol PGS and whether the findings hold consistently in various subgroups. Hence, we could use hazardous drinking as the current alcohol use status as cofounder in our study. However, the inclusion of this covariate might be causing the negative result as it was directly correlated with the PGSs; therefore, analyses with and without this covariate might be worth to be shown, followed by Mendelian randomization. If we used clinical covariates when generating the PGS and again in our regression analyses, that could lead to overfitting with negative consequences in our results. However, we analyzed data using current drinking status as a covariate and found similar results.

The phase of the illness in bipolar disorder is an important issue to be considered while measuring cognitive functions in bipolar disorder. However, unfortunately, we were not able to use manic symptoms in our analysis, as the data did not include an assessment of current manic symptoms.

We used only HapMap3 SNIPs, not all 1000G SNIPs for PGS computation. We did not correct for multiple comparisons (Bonferroni correction). Since most of the confidence intervals did not come close to 1.00, it was obvious that most results would remain non-significant also when these corrections were applied. It might be worth pre-emptying non-significant comparisons.

### 4.5. What Is Already Known on This Subject?

PGS for alcohol dependence is negatively associated with cognition in a normal population.

### 4.6. What Does This Study Add?

Hazardous drinking PGS was not associated with cognitive decline in schizophrenia, schizoaffective, and bipolar disorder outpatients below 70, when a reaction time test and a memory test were used without an assessment of executive functioning and without correcting for manic symptoms.

## 5. Conclusions

Hazardous drinking PGS was not associated with visual memory and reaction time decline in schizophrenia, schizoaffective, and bipolar disorders in outpatients below 70, adjusted with age, age of onset, education, household pattern, and depressive symptoms when a reaction time test and a memory test were used without an assessment of executive functioning and without correcting for manic symptoms, whereas hazardous drinking was associated with better cognition in our previous studies with the same population. It is still unclear whether hazardous drinking PGS is associated with global cognition decline.

The exact reason behind the apparent discrepancy between PGS relations with cognition and behavioral relations with cognition is yet to be discovered. These counterintuitive results question the whole concept of the cognitive impact of alcohol use PGS in psychiatric comorbidities. It also indicates the existence of unknown factors influencing gene-environment interaction warranting further research. In our present study, therefore, any possible findings, whether “positive”, “negative”, or “no” association between hazardous drinking PGS and cognition in these psychiatric conditions, would be important. It is possible to externally validate our research findings; hence, we recommend future replication. Within the SUPER, we are going to explore more details about the cognitive decline associated with schizophrenia, schizoaffective, and bipolar disorder. We are aiming to find out the associating factors for cognitive decline in these psychiatric illnesses.

## Figures and Tables

**Table 1:** Background factors and hazardous drinking PGS in male and female schizophrenia patients.

	Male	Female
	N = 1433	N = 1216
Age (mean (SD))	44.75 (12.32)	46.76 (12.63)
Age of onset (mean (SD))	26.37 (7.74)	27.07 (9.00)
Completing matriculation examination (%)	423 (29.5)	474 (39.0)
Living with spouse (%)	145 (10.1)	297 (24.4)
Having depressive symptoms ^Ω^ (%)	913 (63.7)	790 (65.0)
Currently on psychotropic medications (%)	1403 (97.9)	1194 (98.2)
Currently on antipsychotics (%)	1365 (95.3)	1141 (93.8)
Currently on benzodiazepines (%)	354 (24.7)	318 (26.2)
Currently on antidepressant (%)	483 (33.7)	427 (35.1)
Currently on mood stabilizer (%)	222 (15.5)	241 (19.8)
On some other psychotropics/missing data (%)	22 (1.5)	23 (1.9)
Hazardous drinking PGS (mean (SD))	7.95 × 10^−7^ (9.66 × 10^−7^)	7.63 × 10^−7^ (9.96 × 10^−7^)

SD = Standard Deviation. ^Ω^ MHI-5 cutoff score for depression was ≤72.

**Table 2:** Background factors and hazardous drinking PGS in male and female schizoaffective disorder patients.

	Male	Female
	N = 196	N = 362
Age (mean (SD))	41.53	42.72
Age of onset (mean (SD))	29.52	30.01
Completing matriculation examination (%)	84 (42.9)	167 (46.1)
Living with spouse (%)	40 (20.4)	126 (34.8)
Having depressive symptoms ^Ω^ (%)	140 (71.4)	247 (68.2)
Currently on psychotropic medications (%)	190 (96.9)	353 (97.5)
Currently on antipsychotics (%)	185 (94.4)	336 (92.8)
Currently on benzodiazepines (%)	56 (28.6)	107 (29.6)
Currently on antidepressant (%)	69 (35.2)	148 (40.9)
Currently on mood stabilizer (%)	82 (41.8)	111 (30.7)
On some other psychotropics/missing data (%)	2 (1.0)	4 (1.1)
Hazardous drinking PGS (mean (SD))	7.71 × 10^−7^ (10.29 × 10^−7^)	7.77 × 10^−7^ (9.27 × 10^−7^)

SD = Standard Deviation. ^Ω^ MHI-5 cutoff score for depression was ≤72.

**Table 3:** Background factors and hazardous drinking PGS in male and female bipolar disorder patients.

	Male	Female
	N = 419	N = 706
Age (mean (SD))	45.38 (12.96)	44.41 (12.70)
Age of onset (mean (SD))	36.78 (11.57)	36.05 (11.51)
Completing matriculation examination (%)	143 (34.1)	324 (45.9)
Living with spouse (%)	152 (36.3)	302 (42.8)
Having depressive symptoms ^Ω^ (%)	295 (70.4)	520 (73.7)
Currently on psychotropic medications (%)	397 (94.7)	671 (95.0)
Currently on antipsychotics (%)	345 (82.3)	557 (78.9)
Currently on benzodiazepines (%)	100 (23.9)	196 (27.8)
Currently on antidepressant (%)	124 (29.6)	297 (42.1)
Currently on mood stabilizer (%)	198 (47.3)	263 (37.3)
On some other psychotropics/missing data (%)	11 (2.6)	16 (2.3)
Hazardous drinking PGS (mean (SD))	7.81 × 10^−7^ (9.29 × 10^−7^)	8.17 × 10^−7^ (9.44 × 10^−7^)

SD = Standard Deviation. ^Ω^ MHI-5 cutoff score for depression was ≤72.

**Table 4:** Association of hazardous drinking PGS with RT test and PAL test in male and female schizophrenia patients.

	Male			Female		
RT Test	e^β^ (95% CI)	*p*-Value	R^2^	e^β^ (95% CI)	*p*-Value	R^2^
Median						
Crude	0.97 (0.93, 1.02)	0.212	0.00	1.00 (0.94, 1.05)	0.883	0.00
Adjusted	0.97 (0.93, 1.02)	0.260	0.08	0.99 (0.94, 1.04)	0.599	0.08
SD						
Crude	1.00 (0.95, 1.04)	0.856	0.00	1.00 (0.95, 1.06)	0.931	0.00
Adjusted	1.00 (0.95, 1.05)	0.968	0.10	0.99 (0.95, 1.04)	0.685	0.10
**PAL FTMS**	**β (95% CI)**	***p*** **-Value**	**R^2^**	**β (95% CI)**	***p*** **-Value**	**R^2^**
Crude	0.04 (−0.02, 0.09)	0.121	0.00	−0.05 (−0.11, 0.01)	0.089	0.00
Adjusted	0.04 (−0.01, 0.09)	0.155	0.17	−0.03 (−0.09, 0.02)	0.214	0.18
**PAL TEAS**	**OR (95% CI)**	***p*** **-Value**	**Cohens’ D**	**OR (95% CI)**	***p*** **-Value**	**Cohens’ D**
Crude	1.02 (0.87–1.20)	0.828	0.01	0.90 (0.77, 1.05)	0.190	0.11
Adjusted	1.02 (0.86–1.21)	0.829		0.93 (0.78, 1.11)	0.429	

Adjusted with age, age of onset, education, household pattern, and depressive symptoms. R^2^ = Effect size measures for (simple and multiple) linear regression models. SD = Standard Deviation. OR = Odd Ratio. CI = Confidence Interval. RT = Reaction Time. PAL = Paired Association Learning. FTMS = First Trial Memory Score. TEAS = Total Error Adjusted Score.

**Table 5:** Association of hazardous drinking PGS with RT test and PAL test in male and female schizoaffective disorder patients.

	Male			Female		
RT Test	e^β^ (95% CI)	*p*-Value	R^2^	e^β^ (95% CI)	*p*-Value	R^2^
Median						
Crude	0.94 (0.83, 1.07)	0.356	0.00	0.93 (0.85, 1.02)	0.117	0.00
Adjusted	0.93 (0.82, 1.05)	0.228	0.07	0.94 (0.86, 1.02)	0.129	0.09
SD						
Crude	1.08 (0.94, 1.24)	0.259	0.00	0.96 (0.88, 1.04)	0.266	0.00
Adjusted	1.07 (0.94, 1.21)	0.328	0.10	0.96 (0.89, 1.04)	0.298	0.09
**PAL FTMS**	**β (95% CI)**	***p*** **-Value**	**R^2^**	**β (95% CI)**	***p*** **-Value**	**R^2^**
Crude	−0.10 (−0.24, 0.04)	0.143	0.01	0.08 (−0.03, 0.20)	0.163	0.00
Adjusted	−0.09 (−0.21, 0.03)	0.155	0.28	0.06 (−0.05, 0.17)	0.300	0.11
**PAL TEAS**	**OR (95% CI)**	***p*** **-Value**	**Cohens’ D**	**OR (95% CI)**	***p*** **-Value**	**Cohens’ D**
Crude	0.82 (0.58, 1.15)	0.256	0.21	1.10 (0.84, 1.46)	0.492	0.01
Adjusted	0.85 (0.56, 1.29)	0.456		1.06 (0.79, 1.43)	0.698	

Adjusted with age, age of onset, education, household pattern, and depressive symptoms. R^2^ = Effect size measures for (simple and multiple) linear regression models. SD = Standard Deviation. OR = Odd Ratio. CI = Confidence Interval. RT = Reaction Time. PAL = Paired Association Learning. FTMS = First Trial Memory Score. TEAS = Total Error Adjusted Score.

**Table 6:** Association of hazardous drinking PGS with RT test and PAL test in male and female bipolar disorder patients.

	Male			Female		
RT Test	e^β^ (95% CI)	*p*-Value	R^2^	e^β^ (95% CI)	*p*-Value	R^2^
Median						
Crude	1.01 (0.92, 1.10)	0.872	0.00	1.04 (0.98, 1.10)	0.186	0.00
Adjusted	0.99 (0.91, 1.07)	0.781	0.11	1.04 (0.98, 1.10)	0.208	0.06
SD						
Crude	1.00 (0.91, 1.10)	0.981	0.00	1.02 (0.95, 1.08)	0.649	0.00
Adjusted	0.99 (0.91, 1.07)	0.745	0.15	0.99 (0.94, 1.05)	0.773	0.13
**PAL FTMS**	**β (95% CI)**	***p*** **-Value**	**R^2^**	**β (95% CI)**	***p*** **-Value**	**R^2^**
Crude	−0.03 (−0.15, 0.08)	0.562	0.00	−0.01 (−0.08, 0.07)	0.820	0.00
Adjusted	−0.02 (−0.12, 0.08)	0.679	0.24	0.00 (−0.06, 0.07)	0.935	0.17
**PAL TEAS**	**OR (95% CI)**	***p*** **-Value**	**Cohens’ D**	**OR (95% CI)**	***p*** **-Value**	**Cohens’ D**
Crude	0.93 (0.72, 1.21)	0.590	0.10	0.99 (0.83, 1.19)	0.890	0.01
Adjusted	0.96 (0.71, 1.28)	0.758		1.01 (0.83, 1.21)	0.956	

Adjusted with age, age of onset, education, household pattern, and depressive symptoms. R^2^ = Effect size measures for (simple and multiple) linear regression models. SD = Standard Deviation. OR = Odd Ratio. CI = Confidence Interval. RT = Reaction Time. PAL = Paired Association Learning. FTMS = First Trial Memory Score. TEAS = Total Error Adjusted Score.

## Data Availability

The raw data and materials used for this study are available upon request.

## Supplementary Materials

The following supplementary tables are available online at https://www.mdpi.com/article/10.3390/brainsci11111422/s1, Table S1: Background factors and hazardous drinking PGS in male and female schizophrenia, schizoaffective, and bipolar disorder patients, Table S2: Association of hazardous drinking PGS with RT test and PAL test in male and female schizophrenia, schizoaffective, and bipolar disorder patients.
