# Supplementary material for: Reaction Time and Visual Memory in Connection to Hazardous Drinking Polygenic Scores in Schizophrenia, Schizoaffective Disorder and Bipolar Disorder
Source: Brain Sci. 2021 Oct 27;11(11):1422. doi: 10.3390/brainsci11111422 (PMC8615595; doi:10.3390/brainsci11111422)
Supplement: Supplementary file 1 [file brainsci-11-01422-s001.zip › Supplementary document hazardous drinking PGS.pdf]

brain sciences

MDPI

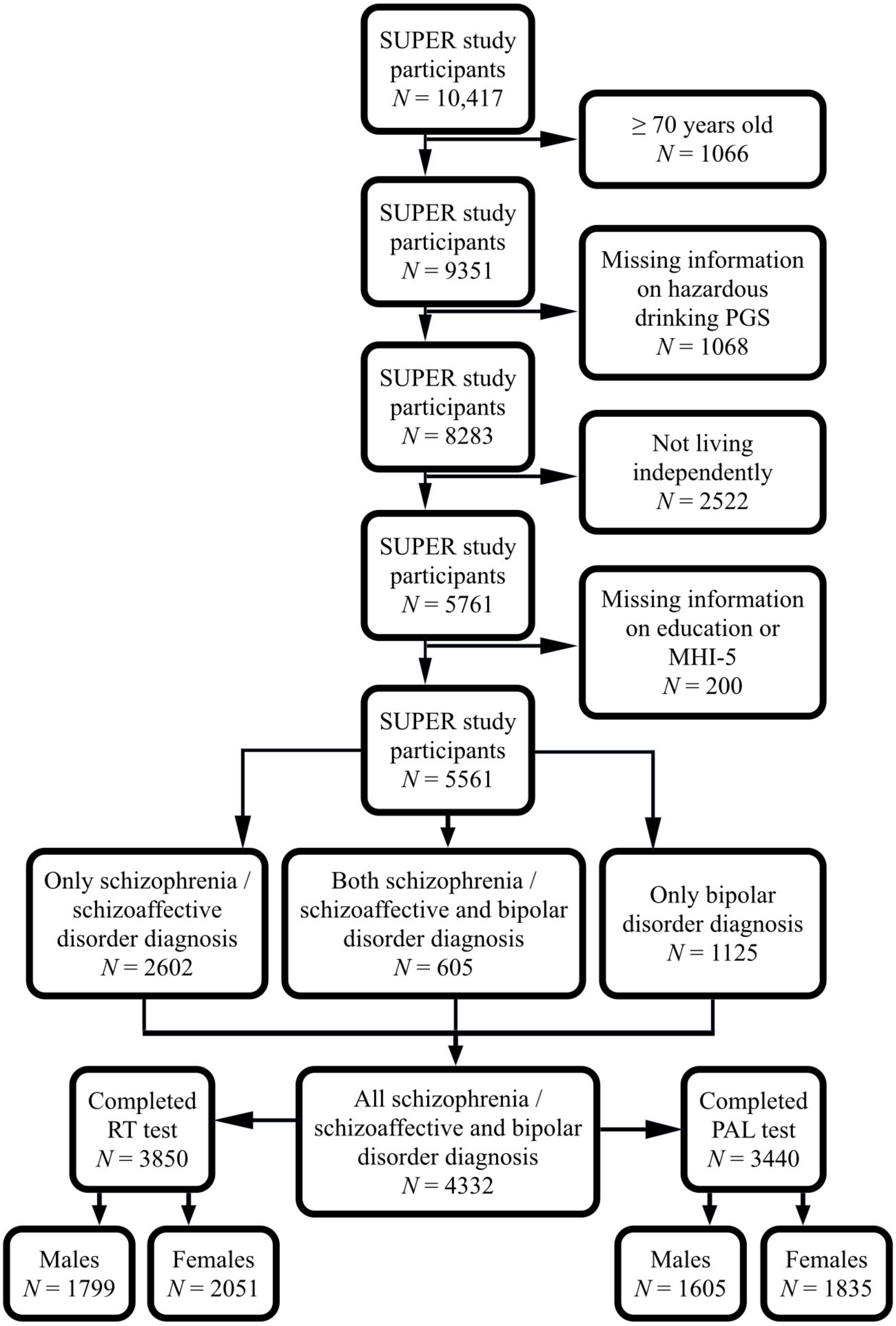

**Supplementary Figure S1.** Flowchart showing the selected study population (combined). SUPER, Suomalainen psykoosisairauksien perinnöllisyysmekanismien tutkimus; MHI-5, mental health inventory-5; RT, reaction time; PAL, paired associative learning.

**Supplementary Figure S2.** Distribution of polygenic scores.

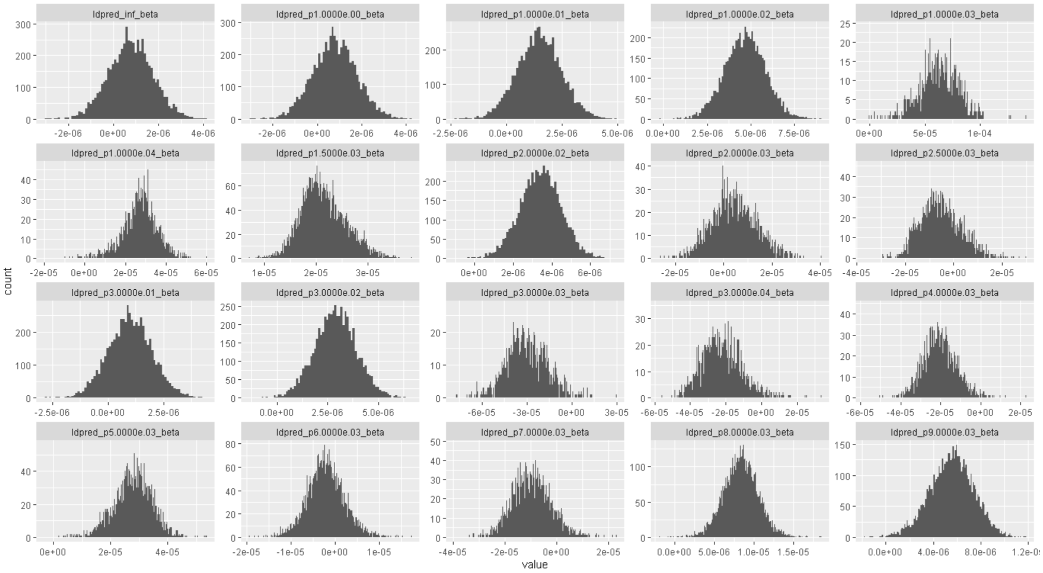


**Supplementary Table S1.** Background factors and hazardous drinking PGS in male and female schizophrenia, schizoaffective and bipolar disorder patients.

| | Male | Female |
|---|---|---|
| | N = 2048 | N = 2284 |
| *Age (mean (SD))* | 44.57 (12.46) | 45.39 (12.64) |
| *Age of onset (mean (SD))* | 26.75 (8.06) | 27.74 (9.38) |
| *Completing matriculation examination (%)* | 650 (31.7) | 965 (42.3) |
| *Living with spouse (%)* | 337 (16.5) | 725 (31.7) |
| *Having depressive symptoms $^{\Omega}$ (%)* | 1348 (65.8) | 1557 (68.2) |
| *Currently on Psychotropic medications (%)* | 1990 (97.2) | 2218 (97.1) |
| *Currently on Antipsychotics (%)* | 1895 (92.5) | 2034 (89.1) |
| *Currently on Benzodiazepines (%)* | 510 (24.9) | 621 (27.2) |
| *Currently on Antidepressant (%)* | 676 (33.0) | 872 (38.2) |
| *Currently on Mood stabilizer (%)* | 502 (24.5) | 615 (26.9) |
| *On some otherPsychotropics/Missing data (%)* | 35 (1.7) | 43 (1.9) |
| *Hazardous drinking PGS (Mean (SD))* | 7.92×10-07 (9.73×10-07) | 7.67 ×10-07 (9.80×10-07) |

SD = Standard deviation. $^{\Omega}$ MHI-5 cutoff score for depression was ≤72.

**Supplementary Table S2.** Association of hazardous drinking PGS with RT test and PAL test in male and female schizophrenia, schizoaffective and bipolar disorder patients.

| | **Male** | | | **Female** | | |
|---|---|---|---|---|---|---|
| **RT test** | **$e^{\beta}$ (95% CI)** | **p-value** | **$R^2$** | **$e^{\beta}$ (95% CI)** | **p-value** | **$R^2$** |
| Median | | | | | | |
| Crude | 0.99 (0.95, 1.03) | 0.494 | 0.00 | 1.00 (0.96, 1.04) | 0.941 | 0.00 |
| Adjusted | 0.97 (0.93, 1.01) | 0.138 | 0.09 | 0.97 (0.93, 1.02) | 0.262 | 0.09 |
| SD | | | | | | |

| | | | | | | |
|---|---|---|---|---|---|---|
| Crude | 1.01 (0.97, 1.06) | 0.585 | 0.00 | 1.00 (0.97, 1.04) | 0.800 | 0.00 |
| Adjusted | 1.01 (0.97, 1.05) | 0.710 | 0.10 | 0.98 (0.94, 1.03) | 0.428 | 0.12 |
| **PAL FTMS** | **β (95% CI)** | **p-value** | **$R^2$** | **β (95% CI)** | **p-value** | **$R^2$** |
| Crude | 0.01 (-0.04, 0.05) | 0.829 | 0.00 | -0.01 (-0.06, 0.03) | 0.557 | 0.00 |
| Adjusted | 0.02 (-0.03, 0.06) | 0.451 | 0.19 | -0.01 (-0.06, 0.03) | 0.549 | 0.17 |
| **PAL TEAS** | **OR (95% CI)** | **p-value** | **Cohens' D** | **OR (95% CI)** | **p-value** | **Cohens' D** |
| Crude | 0.96 (0.85, 1.09) | 0.554 | 0.04 | 0.97 (0.87, 1.08) | 0.604 | 0.03 |
| Adjusted | 0.98 (0.84-1.14) | 0.794 | | 0.97 (0.84, 1.12) | 0.671 | |

Adjusted with age, age of onset, education, household pattern and depressive symptoms. R2= Effect size measures for (simple and multiple) linear regression models. SD= Standard deviation. OR= Odd ratio. CI= Confidence interval. RT= Reaction time. PAL= Paired association learning. FTMS= First trial memory score. TEAS= Total error adjusted score.
